# The Effect of UV Irradiation on the Chemical Structure, Mechanical and Self-Healing Properties of Asphalt Mixture

**DOI:** 10.3390/ma12152424

**Published:** 2019-07-30

**Authors:** Shaopeng Wu, Yong Ye, Yuanyuan Li, Chuangmin Li, Wei Song, Hechuan Li, Chao Li, Benan Shu, Shuai Nie

**Affiliations:** 1 State Key Laboratory of Silicate Materials for Architectures, Wuhan University of Technology, Wuhan 430070, China; 2 Engineering Laboratory of Spatial Information Technology of Highway Geological Disaster Early Warning in Hunan Province, Changsha University of Science & Technology, Changsha 410114, China; 3 Nottingham Transportation Engineering Centre, School of Civil Engineering, University of Nottingham, University Park, Nottingham NG7 2RD, UK; 4 School of Traffic and Transportation Engineering, Changsha University of Science & Technology, Changsha 410114, China

**Keywords:** UV radiation, asphalt mixture, chemical structure, mechanical performance, self-healing performance, CT scanning

## Abstract

Although huge numbers of investigations have been conducted for the ultraviolet (UV) aging of asphalt binder, research rarely focuses on the asphalt mixture. In order to evaluate the aging effect of UV radiation on the asphalt mixture, a dense grade of asphalt mixture was designated and aged by UV radiation for 7, 14 and 28 days respectively. After that, the chemical functional groups of asphalt binder were tested by Fourier transform infrared spectrometer (FTIR). The semi-circular bending strength and fatigue resistance of asphalt concrete were tested to characterize the mechanical properties of the asphalt concrete. To evaluate the self-healing effect of the macro-structure continuity of asphalt concrete intuitively, the computed tomography (CT) scanning machine was used to characterize the crack size of asphalt concrete samples both before and after self-healing. The results show that, with the increase of UV irradiation time, the relative ratios of the C=O and S=O bands’ areas of recovered asphalt binder increase significantly. UV radiation can significantly weaken the mechanical and self-healing properties of asphalt mixture, making the asphalt mixture to have worse macro-structure continuity, lower failure strength and worse fatigue resistance. Moreover, the longer the UV irradiation time is, the degradation effect of UV radiation on asphalt mixture becomes more obvious.

## 1. Introduction

For the complex work condition of asphalt pavement, there are many factors that can affect the service span, such as the vehicle, temperature [1], water and UV radiation [2,3,4]. Therefore, in general, the service time of asphalt pavement is always shorter than its designated life. UV radiation has attracted more and more focus for its significant aging effects on the asphalt materials [5]. After UV aging, the elemental ratios and chemical components of asphalt binder are changed [6], which results in the deterioration of physical and rheological performance of asphalt binder and the pavement performance of its concrete [7,8,9]. Especially for the areas with high intensity of UV radiation, the aging effect is more obvious [10].

In the previous work [11], the researchers conducted much work on the UV aging mechanism and aging effects on the technical performance of asphalt materials. For instance, Vallerga [12] used UV radiation and infrared light to irradiate the asphalt film in a film oven respectively and the results indicated that UV radiation could significantly affect the softening point and ductility of asphalt binder, therefore the UV radiation was an important factor causing the aging phenomenon of asphalt binder. Zhang [13] studied the aging effect of three types of aging methods namely thin film oven test (TFOT), pressure aging vessel (PAV), and UV radiation, the results showed that the UV aging and PAV aging have more obvious effects on the degradation of styrene–butadiene–styrene (SBS) polymer. Glotova [14] studied the effect of UV aging on asphalt binder by testing the chemical properties, structures and composition contents of asphalt binder before and after aging, and found that UV radiation has obvious degeneration effects on these properties, and the type of light irradiation also has a close relationship with the photo-oxidation aging speed. Wu et al. [15] used a high-pressure mercury lamp to irradiate both a base asphalt binder and a polymer modified asphalt binder; the results of the Fourier infrared test and dynamic shear rheometer (DSR) test also showed that UV radiation can obviously age the asphalt binder, and different intensities of UV radiations caused different aging effects. The above research verifies the degradation effects of UV radiation on the technical performance of asphalt materials, and it is truly important to investigate the degradation behaviors of this.

Compared with the asphalt binder, there was less work focused on the UV aging effect of UV radiation on the pavement performance asphalt mixture. In fact, the UV radiation always directly affects the asphalt mixture during the service period of pavement [16], so conducting the UV aging test on the asphalt mixture is better to simulate the true service situation of the asphalt road. In this research, a dense grade of asphalt mixture with the nominal maximum aggregate size of 13.2 mm (AC-13) was designated, and aged by UV radiation for 7, 14 and 28 days respectively. After UV aging, the mechanical performance of asphalt concrete was tested by a semi-circle bending test and the anti-fatigue properties of asphalt mixture were investigated by three-point bending fatigue test.

On the other hand, basing on Reference [17], it can be found that the thermal oxidative aging (85 °C for 240 h) can decrease the mechanical performance and healing levels of asphalt concrete obviously. Although the mechanism of thermal oxidative aging of asphalt material is not at all the same with UV aging, the effect tendency on the performance is somehow the same [18], so that UV aging may also cause a significant effect on the self-healing performance of asphalt concrete. In this research, the chemical functional groups of asphalt binder were tested by Fourier transform infrared spectrometer (FTIR). The healing index of the mechanical performance of asphalt concrete was investigated to characterize the self-healing performance. Meanwhile, a computed tomography (CT) scanning machine was also used to quantitatively test the crack size of asphalt concrete before and after self-healing. The results can be used to express the degradation behaviors of the chemical structure, mechanical and self-healing properties of asphalt mixture exposed during UV irradiation.

## 2. Materials and Experimental Methods

### 2.1. Materials

The most commonly used asphalt mixture with the nominal maximum aggregate size of 13.2 mm (AC–13) was designated for the UV aging simulation test. The asphalt binder used in this research was base asphalt with penetration grade of 60/80. The physical performance and dynamic viscosity of asphalt binder are shown in Table 1.

**Figure 1:**
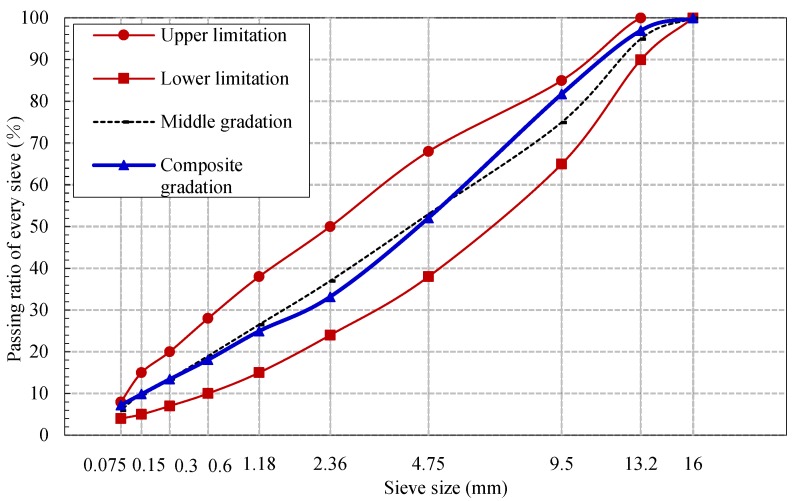
Composite aggregate grading of the asphalt mixture.

The mix ratio of asphalt mixture was ensured with the Marshall method. The optimum oil–aggregate ratio of the asphalt mixture is 4.7%. The ratios of different grades of aggregate were 13.2–16.0 mm:9.5–13.2 mm:4.75–9.5 mm:2.36–4.75 mm:0–2.36 mm:filler = 3:15:33:13:32:4. The aggregate was basalt and the filler was the fine powder of limestone. The composite aggregate grading of the asphalt mixture is given in Figure 1. The volume parameters of asphalt concrete are shown in Table 2.

### 2.2. Experimental Methods

#### 2.2.1. Parameter Design of UV Aging Test

**Figure 2:**
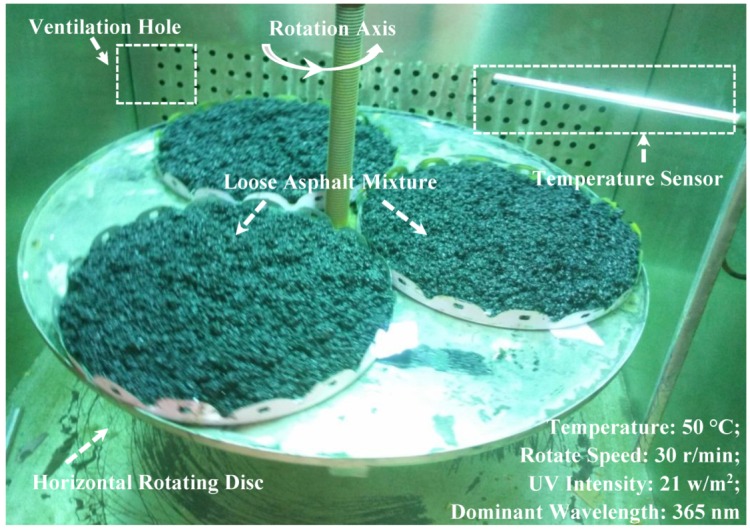
UV aging test of loose asphalt mixtures.

The irradiation intensity of UV light used in the UV aging simulation test of asphalt mixture was 21 w/m^2^, which was produced by four UV lamps, the power of every UV lamp was 500 W. The aging time was 7 days, 14 days and 28 days respectively. The wavelength range of the UV radiation was from 200 nm to 400 nm. The temperature of chamber of the UV aging simulation instrument was 25 °C, it was tested by the temperature sensor in the chamber. The surface temperature of the asphalt mixture was about 50 °C, it was tested by an infrared temperature tester (SMART SENSOR AR-300+, SMART SENSOR, Hongkong, China). According to the thermal oxidative aging test (short term aging) of asphalt mixture, the spread density of asphalt mixture was 22 kg/m^2^. During the UV aging test, the asphalt mixture was mixed every 24 h. Figure 2 shows the UV aging test of loose asphalt mixtures.

#### 2.2.2. Recovery of Asphalt Binder from Asphalt Mixtures

After different times of UV irradiation, the asphalt binders were recovered from the asphalt mixtures by dissolution-centrifugal separation method. The solvent was trichloroethylene (C_2_HCl_3_), the purity of which was higher than 99%. The detailed procedure was as follows. First, the asphalt mixture was placed into C_2_HCl_3_ for half an hour, and then the centrifugal separation method was used to get the asphalt–C_2_HCl_3_ solution; this step was repeated 2–3 times. Finally, the asphalt–C_2_HCl_3_ solution was placed in the fume hood and dried naturally for 72 h.

**Figure 3:**
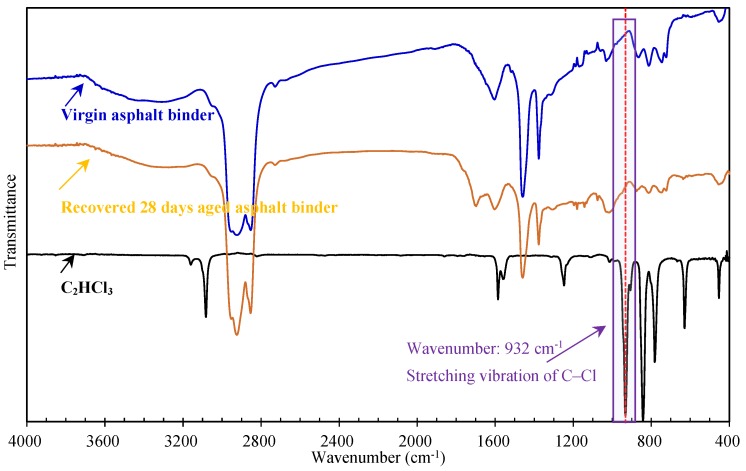
FTIR spectra of the pure asphalt binder, recovered 28 days aged asphalt binder and C_2_HCl_3._

In order to detect whether the C_2_HCl_3_ would affect the Fourier transform infrared spectrometer (FTIR, Nexus, Thermo Nicolet, Columbus, OH, USA) result of recovered asphalt binder, after 72 h volatilization and drying, the chemical structures of the virgin asphalt binder, recovered 28 days aged asphalt binder and C_2_HCl_3_ were tested by FTIR respectively. The results are shown in Figure 3. From Figure 3, a special absorption band at the wavenumber of 932 cm^−1^ can be observed in the FTIR spectrum of C_2_HCl_3_, it is caused by the stretching vibration of C–Cl of C_2_HCl_3_. This absorption band was very obvious and could be found in the FTIR spectrum of the virgin asphalt binder. Therefore, it could be used to test whether the C_2_HCl_3_ volatilized completely by comparing the FTIR spectra differences of the virgin asphalt binder and recovered 28 days aged asphalt binder. It was found that, in the FTIR spectrum of 28 days aged asphalt binder, there was no absorption band at wavenumber 932 cm^−1^, therefore the C_2_HCl_3_ volatilized completely after 72 h volatilization (at least, it did not obviously affect the FT-IR of recovered asphalt binder).

#### 2.2.3. Chemical Functional Group Test of Recovered Asphalt Binders

The chemical structures of recovered asphalt binders were characterized by FTIR, the procedure of the FTIR test was as follows. The asphalt was dissolved in carbon disulfide (CS_2_), the concentration of the asphalt binder was 5.0 wt%; then the asphalt–CS_2_ solution was dropped on a potassium bromide (KBr) window, and the potassium bromide window was set under an incandescent light bulb until the CS_2_ volatized completely. The sweep times was 32, the sweep range of wavenumber was from 4000 cm^−1^ to 400 cm^−1^.

#### 2.2.4. Mechanical Properties Tests of Asphalt Concrete

The mechanical properties tests of asphalt concretes before and after UV aging were investigated by both the semi-circular bending test and three-point bending fatigue test.

**Figure 4:**
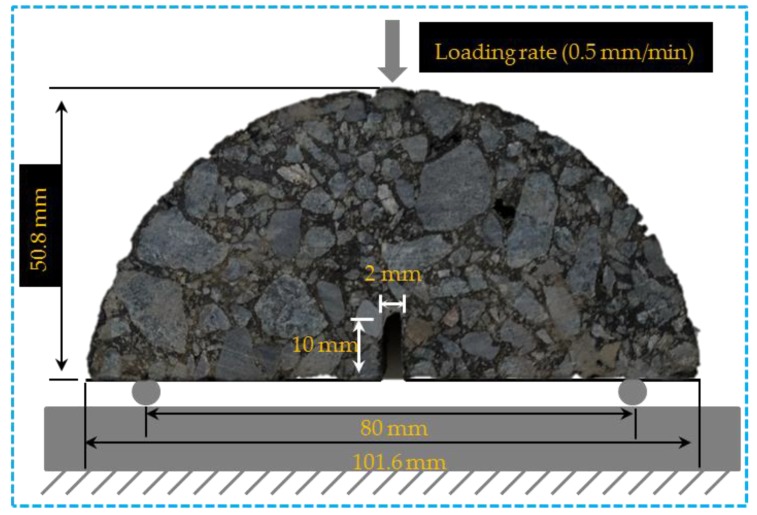
Schematic diagram of the semi-circular bending test of asphalt concrete.

The machine used for the semi-circular bending test was UTM-25 (IPC, Sydney, Australia). The diameter of the sample was 100 mm, the thickness was 50 mm. In order to control the crack position during the test, a notch with depth of 10 mm and width of 2 mm was precut. The testing temperature was −10 °C, and the loading speed was 0.5 mm/min. The schematic diagram of semi-circular bending test of asphalt concrete is shown in Figure 4.

The fatigue tests of asphalt concretes were tested at both 0.4 and 0.6 stress ratios, which was conducted with UTM-25 machine as well. The testing temperature was 25 °C and the Poisson ratio was designated as 0.35. A semi-sine wave of loading was used, and the loading frequency was 1.0 Hz (loading time was 0.1 s, and interval was 0.9 s). To control the temperature of the samples, all of them were put in the UTM chamber under constant temperature (25 °C) for more than five h. For the same aging status, three samples of asphalt concretes were tested to calculate the average values and standard deviations of the fatigue lives.

#### 2.2.5. Self-Healing Performance Tests of Asphalt Concrete

The semi-circular bending strength and fatigue life of initial asphalt concrete and asphalt concrete after self-healing were tested respectively, which was to obtain the healing index of asphalt concrete. A higher value of healing index expresses the better self-healing performance of asphalt concrete, and vise versa.

**Figure 5:**
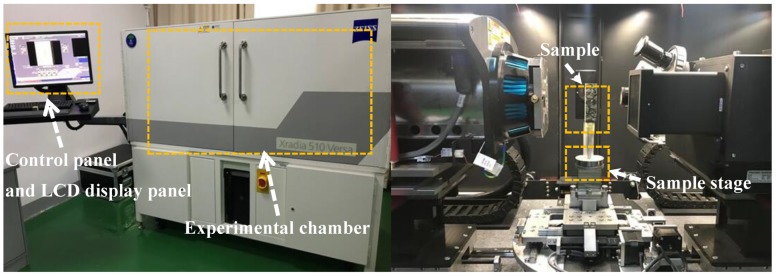
Experimental diagram of the computed tomography (CT) scanning test.

Meanwhile, a CT scanning machine (Xradia 510 Versa, ZEISS, Oberkochen, Germany) was used to characterize the crack size of asphalt concrete samples before and after self-healing. Figure 5 shows the diagram of this test. The real spatial resolution of the machine was 0.7 microns, and the voxel size was as low as 70 nanometers. The self-healing time of the samples was 72 h, and the temperature in the environmental chamber was 50 °C.

### 2.3. Logic Map of Experimental Design

**Figure 6:**
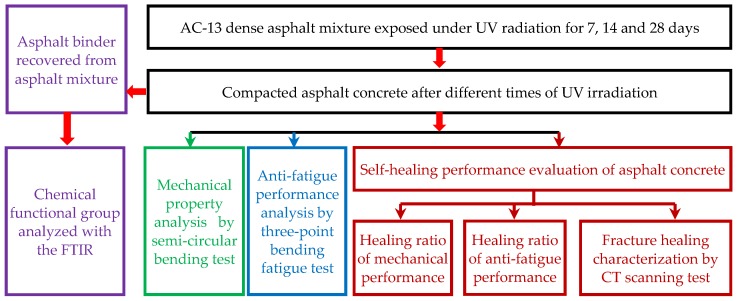
Logic map of the experimental design.

The logic map of experimental design is shown in Figure 6.

## 3. Results and Discussions

### 3.1. Characterization of the UV Aging Status of Recovered Asphalt Binder

The atoms that form the chemical bonds or functional group of organic molecules are always in a constant state of vibration, and the vibration frequency of that is equal to the vibration frequency of the infrared light [23]. When the infrared light irradiates on the organic molecules, the vibrational absorption of infrared light can be observed in the chemical bonds or functional groups in molecules [24]. The different chemical bonds or functional group have different absorption frequencies, the infrared spectrum will be reflected in different positions, thus the FTIR can be used to characterize the chemical bonds or functional molecules in organic molecules [25]. Because of the irradiation of UV light, the chemical groups such as C=C and C–H without oxygen can be oxidized, and form oxygen-containing functional groups, such as the carbonyl group (C=O) and sulfoxide functional group (S=O) [26]. Therefore, the content of C=O and S=O will increase after UV aging, the absorption band areas of these two chemical functional groups will increase as well, which can be used to characterize the aging status of asphalt binder. The bigger the absorption band areas of C=O and S=O, the more serious the UV aging status.

**Figure 7:**
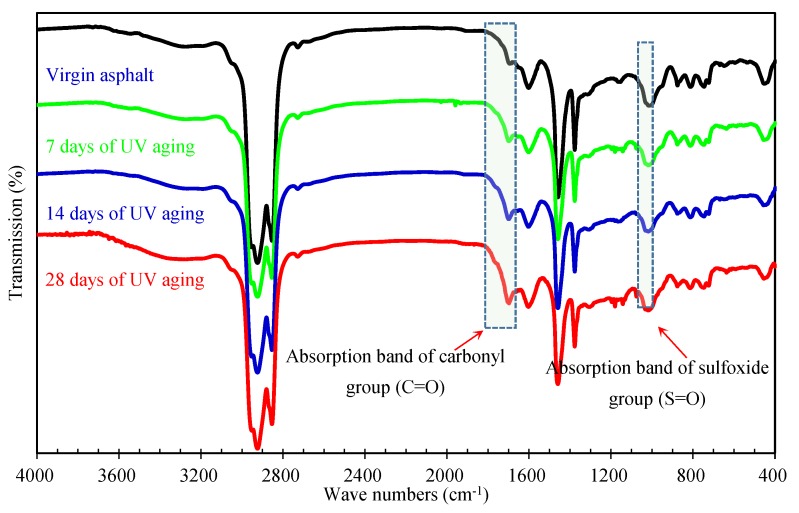
FTIR spectra of asphalt binders recovered from aged mixtures.

The FTIR spectra of asphalt binders recovered from aged mixtures are shown in Figure 7. From Figure 7, the absorption bands of the main chemical functional groups of asphalt binder were in the wavenumber range of 600–2000 cm^−1^, the absorption bands at 1700 cm^−1^ and 1030 cm^−1^ were caused by the C=O and S=O respectively. However, the absorption bands of the C=O and S=O can also be observed in FTIR spectra of original asphalt binder. This is because thermo–oxidative aging happens during the production and storage of asphalt binder, and the mixing of asphalt mixture. From Figure 7, the absorption bands C=O and S=O increased with the increasing aging time, indicating the more serious aging status of asphalt binders recovered from asphalt mixtures under longer times of UV irradiation.

To quantificationally characterize the aging status of UV aging status of recovered asphalt binder, the relative ratios of the areas of C=O absorption band and S=O absorption band were calculated based on Equations (1) and (2) respectively.

(1) $$\mathrm{CRR}=\frac{S_{1700{\;\mathrm{cm}}^{-1}}}{\sum S_{2000{\;\mathrm{cm}}^{-1}\;\sim \;600{\;\mathrm{cm}}^{-1}}},$$

(2) $$\mathrm{SRR}=\frac{S_{1030{\;\mathrm{cm}}^{-1}}}{\sum S_{2000{\;\mathrm{cm}}^{-1}\;\sim \;600{\;\mathrm{cm}}^{-1}\;}},$$

where, the (CRR) and (SRR) are the C=O relative ratio and S=O relative ratio respectively, $S_{1700{\;\mathrm{cm}}^{-1}}$ and $S_{1030{\;\mathrm{cm}}^{-1}}$ are the areas of C=O absorption band and S=O absorption band respectively, $\sum S_{2000{\;\mathrm{cm}}^{-1}\;\sim \;600{\;\mathrm{cm}}^{-1}}$ is the sum of areas of all bands in the wavenumber range of $2000{\;\mathrm{cm}}^{-1}\;\sim \;600{\;\mathrm{cm}}^{-1}$.

The relative ratios of the areas of C=O and S=O absorption bands are shown in Table 3. The bigger the CRR and SRR values are, the more serious the aging status of asphalt binder is [27]. From Table 3, after 7 days, 14 days and 28 days of UV irradiation, the CRR value of which increased by 128.6%, 226.2% and 358.3% respectively, the SRR value of which increased by 21.7%, 34.5% and 40.4% respectively. The CRR and SRR values of recovered asphalt binder increased significantly with the increase of UV irradiation time, the content of oxygen-containing functional groups in asphalt binder increased obviously as well, indicating that the aging status of asphalt binder was more serious with longer UV irradiation time.

### 3.2. UV Radiation Effects on Mechanical Properties of Asphalt Concrete

#### 3.2.1. UV Radiation Effects on the Semi-Circular Bending Strength of Asphalt Concrete

Asphalt concrete is granular in nature, and its macroscopic behavior is mainly a function the interactions of asphalt binder and aggregate and the UV aging of asphalt binder may significantly influence the mechanical property of asphalt concrete [28,29]. The semi-circular bending test was conducted on the asphalt concrete before and after UV aging. Three parallel tests were performed at each aging time, the bending strength of asphalt concrete was calculated according to Equation (3), the average bending strength of these three specimens were taken as the final result.

(3) $$S_{t}=\frac{2\cdot P}{\pi \cdot d\cdot h}$$

where, *S_t_* is the semi-circular bending strength of asphalt concrete, MPa; *P* is the maximum loading of every specimen, N; *d* is the diameter of every specimen, mm; *h* is the thickness of every specimen, mm.

**Figure 8:**
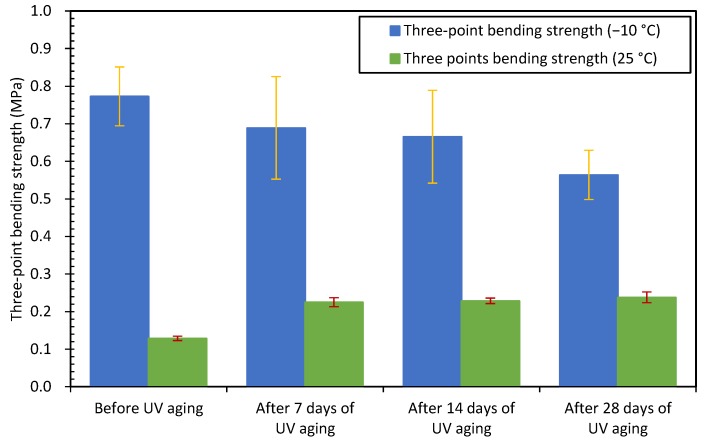
Semi-circular bending strengths of asphalt concretes before and after UV aging.

Figure 8 gives the average bending strengths of all asphalt concretes. From Figure 8, when after 7, 14 and 28 days of UV aging, the semi-circular bending strengths of asphalt concretes at −10 °C decreased by 10.8%, 15.6% and 31.4% respectively; while the semi-circular bending strengths of asphalt concretes at 25 °C increased by 97.6%, 110.0% and 118.6% respectively. Therefore, after different times of UV aging, the semi-circular bending strengths of asphalt concretes at −10 °C tended to decrease, while they showed an opposite tendency at 25 °C. With the extension of UV exposure time, the tendencies were more obvious. The reason is that, after aging the asphalt binder tends to be stiffer and harder, the asphalt binder is inherently brittle at low temperature condition (−10 °C); the comprehensive action of UV aging and low temperature causes the degradation of the three bending strengths of asphalt concretes. However, at relative high temperature (25 °C), the harder effect of UV aging on asphalt binder can partially offset the softening effect of high temperature, therefore the 25 °C semi-circular bending strengths of asphalt concretes were increased. This results also the same as other research work [30].

#### 3.2.2. UV Radiation Effects on the Fatigue Resistance of Asphalt Concrete

**Figure 9:**
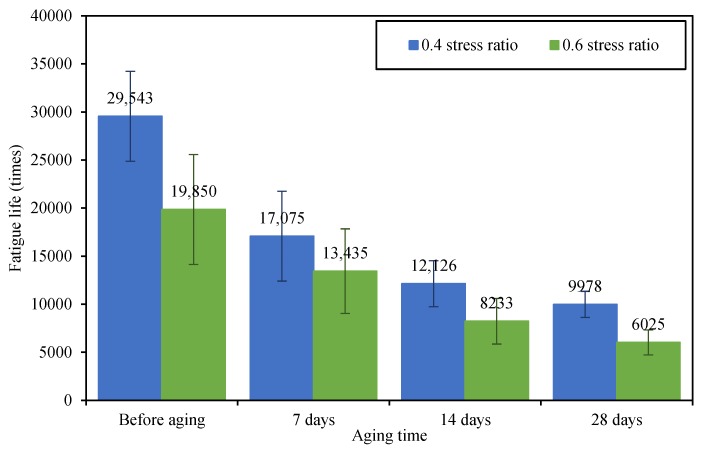
Fatigue lives of asphalt concrete under different stress ratios.

The fatigue tests of asphalt concretes before and after UV aging were conducted at 0.4 and 0.6 stress ratios respectively. The results are shown in Figure 9.

It can be found from Figure 9 that, after UV aging, the fatigue lives of asphalt concretes at both 0.4 and 0.6 stress ratios decreased, and with prolonging UV irradiation time, the decreased range was more significant. In detail, after being aged for 7, 14 and 28 days, the fatigue lives of asphalt concrete under 0.4 stress ratio of loading decreased by 42.2%, 58.9% and 66.2% respectively, and under 0.6 stress ratio of loading decreased by 32.3%, 58.2% and 69.6% respectively. Therefore, UV radiation decreased the fatigue resistance of asphalt concretes; the longer the UV irradiation time was, the more significant the aging effect.

### 3.3. UV Aging Effects on the Self-Healing Performance of Asphalt Concrete

#### 3.3.1. Healing Percentages of Semi-Circular Bending Strengths

**Figure 10:**
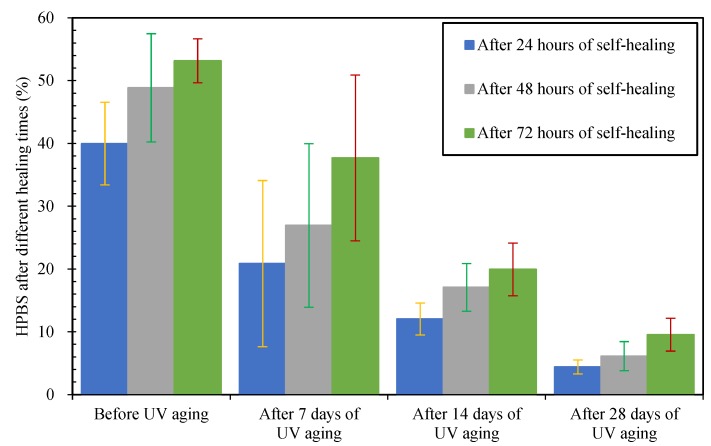
Healing percentages of −10 °C bending strengths after different healing times.

**Figure 11:**
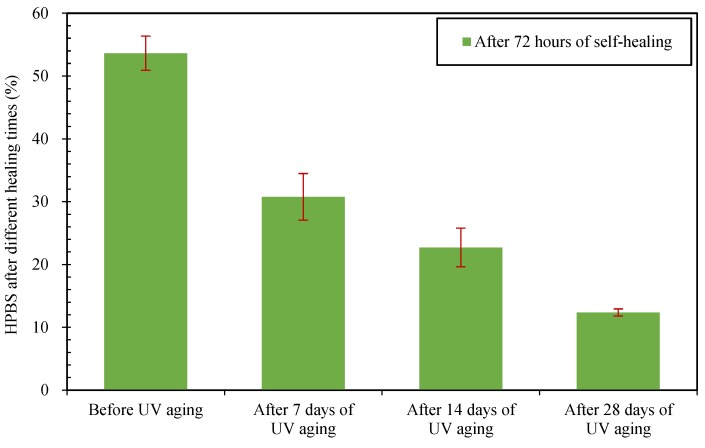
Healing percentages of 25 °C bending strengths after 72 h of self-healing.

The healing percentages of semi-circular bending strengths of asphalt concretes were calculated according to Equation (4). The healing percentages of semi-circular bending strengths (HPBS) values of asphalt concretes before and after UV irradiation are listed in Figure 10 and Figure 11.

(4) $$\mathrm{HPBS}=\frac{BS_{after\;aging}}{BS_{before\;aging}}\times 100\%$$

where, the HPBS is the healing percentages of semi-circular bending strengths of asphalt concretes, %; *BS_before aging_* and *BS_after aging_* are the healing percentages of semi-circular bending strengths of asphalt concretes before and after UV aging respectively, MPa.

From Figure 10, for the −10 °C bending strengths, the self-healing effects of asphalt concretes had time sensitivity: the HPBS values of asphalt concretes increased with the increase of healing time. When the healing times were 24 h and 72 h, the HPBS values of asphalt concretes before UV aging were 40.0% and 53.2% respectively. Compared with asphalt concrete without UV irradiation, after self-healing, the HPBS values of asphalt concretes after 7, 14 and 28 days UV irradiation decreased significantly; the HPBS decrement of asphalt concretes after different times of UV irradiation are listed in detail in Table 4. From Figure 11 and Table 4, after 72 h of self-healing, the same tendency could also be observed for the 25 °C bending strengths of asphalt concrete. Therefore, the UV irradiation could weaken the self-healing performance of asphalt concrete obviously, and the longer the irradiation time was, the worse the self-healing performance of asphalt concrete.

#### 3.3.2. Healing Percentages of Fatigue Performance

Initial fatigue lives and the fatigue lives after self-healing of asphalt concretes are shown in Table 5. From Table 5, after UV aging, the fatigue lives of all asphalt concretes before and after healing decreased obviously, with the increase of aging time, the reductions were more significant.

To quantificationally compare the abilities of the self-healing performance of asphalt concretes, the healing percentages of fatigue lives (HPFL) were calculated according to Equation (5).

(5) $$\mathrm{HPFL}=\frac{FL_{after\;aging}}{FL_{before\;aging}}\times 100\%$$

where, the HPFL is the healing percentages of fatigue lives of asphalt concretes, %; *FL_before aging_* and *FL_after aging_* are the healing percentages of fatigue lives of asphalt concretes before and after UV aging respectively, times.

**Figure 12:**
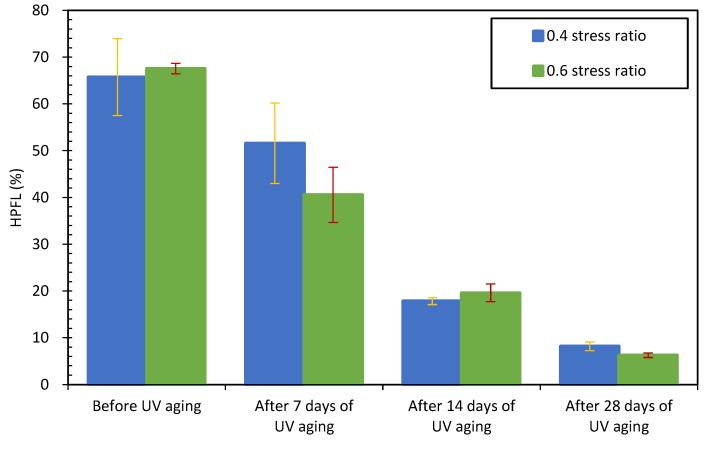
HPFL values of asphalt concretes after different aging times.

The HPFL values of asphalt concrete after different times of UV irradiation are listed in Figure 12. From Figure 12, for the 0.4 stress ratio, the HPFL values of asphalt concretes before UV aging was 65.7%, it decreased to 21.4%, 72.9% and 87.5% after 7, 14, and 28 days of UV irradiation respectively; for the 0.6 stress ratio, the HPFL values of asphalt concretes also showed a decreasing tendency. The lower HPFL values meant a worse self-healing effect, so the self-healing effect of asphalt concretes after UV irradiation decreased, the self-healing performance of asphalt concrete decreased with increasing UV irradiation time. The reason is that the self-healing of asphalt concrete is accomplished mainly based on two patterns, firstly, due to the drain of asphalt binder into the cracks [31], it can fill the cracks and recover the structural continuity of asphalt concrete [32]; secondly, the thermal expansion of asphalt binder also plays an important role in the self-healing of asphalt concrete [33]. However, after UV aging, the viscosity of asphalt binder in its concrete is much higher than that of before aging [26]. At the same temperature, the asphalt binder in asphalt concrete after UV aging flows more slowly than that of initial asphalt concrete, the relative high viscosity limits the flow of asphalt binder [34,35,36], therefore results in a lower self-healing ratio.

#### 3.3.3. Fracture Healing Characteristics Analysis by CT Scanning Test

**Figure 13:**
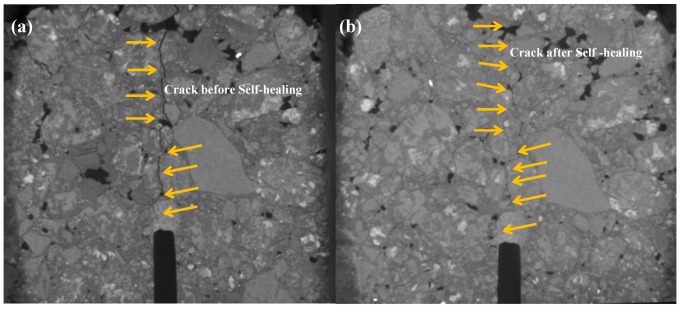
CT scanning figures of initial asphalt concretes (**a**) before self-healing; (**b**) after self-healing.

**Figure 14:**
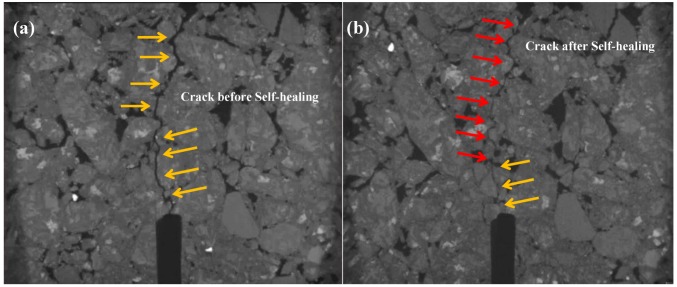
CT scanning figures of asphalt concretes after UV irradiation (**a**) before self-healing; (**b**) after self-healing.

The CT scanning figures of asphalt concretes before and after UV irradiation are shown in Figure 13 and Figure 14 respectively, where the self-healing effect of the cracks in the asphalt concrete intuitively can be observed. From Figure 13, the asphalt concrete had an obvious crack before self-healing, while, after self-healing, the crack almost disappeared, and the crack could not be found unless we observed very carefully, which indicated that the crack healed very well. From Figure 14, after self-healing, the crack of asphalt concrete after 28 days of UV irradiation also decreased obviously, especially the lower part of sample almost healed completely, but a small crack could still be observed in the upper part (marked in Figure 14 by the red arrow), the continuity of macro structure of asphalt concrete was not completely repaired. Therefore, despite being under the same self-healing condition, the self-healing effect of asphalt concrete before UV irradiation was much better than that of asphalt concrete after 28 days of UV irradiation. The results indicated that the asphalt concrete after UV irradiation still had a certain self-healing ability, but the self-healing ability of which was reduced obviously during UV irradiation, therefore UV radiation can significantly decrease the self-healing performance of asphalt concrete.

## 4. Conclusions

In this research, the AC-13 asphalt mixture was exposed under UV radiation for 7, 14 and 28 days to investigate the degradation behaviors of the chemical structure, mechanical and self-healing properties of asphalt mixture exposed under the UV radiation, the flowing conclusions can be obtained.

(1) With the increase of UV irradiation time, the CRR and SRR values of recovered asphalt binder increased significantly, meanwhile, the fatigue life of asphalt concrete under both the 0.4 and 0.6 stress ratios decreased gradually, indicating that the UV radiation could weaken the chemical structure and fatigue resistance of asphalt concrete.
(2) UV radiation significantly affected the asphalt semi-circular bending strength of asphalt concrete, it decreased the −10 °C bending strength of asphalt concrete, while increased the 25 °C bending strength, and the longer the exposure duration was, the more obvious the aging effect.
(3) After UV aging, the HPBS and HPFL values of asphalt concrete decreased obviously; the CT scanning figures also showed the same tendency with HPBS and HPFL values, under the same self-healing condition. The self-healing effect of the crack in the asphalt concrete after UV irradiation was worse than that of initial asphalt concrete—a crack could still be observed in the asphalt concrete after UV irradiation. The results indicated the UV radiation could significantly reduce the self-healing performance of asphalt concrete, causing a worse macro-structure continuity, lower failure strength and worse fatigue resistance.

## Figures and Tables

**Table 1:** Physical performance and dynamic viscosity of asphalt binder.

Technical Parameter	25 °C Penetration (0.1 mm)	Softening Point (°C)	10 °C Ductility (cm)	60 °C Dynamic Viscosity (Pa·s)
Result	66.3	51.0	165	302.500
Standard	ASTM D5 [19]	ASTM D36 [20]	ASTM D113 [21]	ASTM D4402 [22]

**Table 2:** Volume parameters of asphalt concrete.

Volume Parameters	Theoretical Maximum Specific Gravity	Bulk Volume Relative Density	VV(%)	VMA(%)	VFA(%)
Results	2.676	2.556	4.5	14.5	69.0

**Table 3:** Carbonyl relative ratio (CRR) and sulfoxide relative ration (SRR) of asphalt recovered from aged asphalt mixtures.

Aging Time	C=O		S=O	
	CRR Value	Relative Change to Original State (%)	SRR Value	Relative Change to Original State (%)
Virgin asphalt	0.0084	-	0.0750	-
7 days	0.0192	128.6	0.0913	21.7
14 days	0.0274	226.2	0.1009	34.5
28 days	0.0385	358.3	0.1053	40.4

**Table 4:** HPBS decrement of asphalt concretes after different times of UV irradiation (%).

Aging Time	HPBS Decrement at −10 °C			HPBS Decrement at 25 °C
	24 h	48 h	72 h	72 h
7 days	47.7	44.8	29.6	42.5
14 days	70.0	64.9	62.5	57.6
28 days	89.0	87.5	82.1	76.8

**Table 5:** Initial fatigue lives and the fatigue lives after self-healing of asphalt concretes.

Stress Ratio	Status	Before Aging	7 days	14 days	28 days
0.4 stress ratio	Initial fatigue life	29543	17075	12126	9978
	After self-healing	19352	8703	2156	819
0.6 stress ratio	Initial fatigue life	19850	13435	8233	6025
	After self-healing	13413	5360	1611	379

## References

[1] Bazzaz M., Darabi M.K., Little D.N., Garg N. (2018). A straightforward procedure to characterize nonlinear viscoelastic response of asphalt concrete at high temperatures. Transp. Res. Rec..

[2] Zeng W., Wu S., Wen J., Chen Z. (2015). The temperature effects in aging index of asphalt during UV aging process. Constr. Build. Mater..

[3] de Sá M.D., Lins V.D., Pasa V.M., Leite L.F. (2013). Weathering aging of modified asphalt binders. Fuel Process. Technol..

[4] Fernández-Gómez W.D., Rondón Quintana H., Reyes Lizcano F. (2013). A review of asphalt and asphalt mixture aging: Una revisión. Ing. Investig..

[5] Li R., Xiao F., Amirkhanian S., You Z., Huang J. (2017). Developments of nano materials and technologies on asphalt materials–A review. Constr. Build. Mater..

[6] Li Y., Wu S., Liu Q., Xie J., Li H., Dai Y., Li C., Nie S., Song W. (2019). Aging effects of ultraviolet lights with same dominant wavelength and different wavelength ranges on a hydrocarbon-based polymer (asphalt). Polym. Test..

[7] Liu X., Wu S., Liu G., Li L. (2015). Effect of ultraviolet aging on rheology and chemistry of LDH-modified bitumen. Materials.

[8] Zhang C., Yu J., Xu S., Xue L., Cao Z. (2016). Influence of UV aging on the rheological properties of bitumen modified with surface organic layered double hydroxides. Constr. Build. Mater..

[9] Zhang H., Zhu C., Kuang D. (2015). Physical, rheological, and aging properties of bitumen containing organic expanded vermiculite and nano-zinc oxide. J. Mater. Civ. Eng..

[10] Xiao F., Amirkhanian S.N., Karakouzian M., Khalili M. (2015). Rheology evaluations of WMA binders using ultraviolet and PAV aging procedures. Constr. Build. Mater..

[11] Lins V., Araújo M., Yoshida M., Ferraz V., Andrada D., Lameiras F. (2008). Photodegradation of hot-mix asphalt. Fuel.

[12] Vallerga B., Monismith C., Granthem K. (1957). A study of some factors influencing the weathering of paving asphalts. Assoc Asphalt Paving Technol Proc.

[13] Zhang D., Zhang H., Shi C. (2017). Investigation of aging performance of SBS modified asphalt with various aging methods. Constr. Build. Mater..

[14] Glotova N., Kats B., Gorshkov V. (1974). Photooxidation of asphalts in thin films. Chem. Technol. Fuels Oils..

[15] Wu S., Pang L., Liu G., Zhu J. (2010). Laboratory study on ultraviolet radiation aging of bitumen. J. Mater. Civ. Eng..

[16] Li Y., Wu S., Dai Y., Pang L., Liu Q., Nie S., Li H., Wang Z. (2018). Laboratory and field evaluation of sodium stearate organically modified LDHs effect on the anti aging performance of asphalt mixtures. Constr. Build. Mater..

[17] Norambuena-Contreras J., Yalcin E., Garcia A., Al-Mansoori T., Yilmaz M., Hudson-Griffiths R. (2018). Effect of mixing and ageing on the mechanical and self-healing properties of asphalt mixtures containing polymeric capsules. Constr. Build. Mater..

[18] Wu S., Zhao Z., Li Y., Pang L., Amirkhanian S., Riara M. (2017). Evaluation of aging resistance of graphene oxide modified asphalt. Appl. Sci..

[19] ASTM D. (1992). Standard test method for penetration of bituminous materials. Annual Book of ASTM Standards USA.

[20] Standard A. (2009). D36. Standard Test Method for Softening Point of Bitumen (Ring-and-Ball Apparatus).

[21] ASTM (1979). Standard Test Method for Ductility of Bituminous Materials.

[22] ASTM (2012). Standard Test Method for Viscosity Determination of Asphalt at Elevated Temperatures Using a Rotational Viscometer.

[23] Zhong K., Cao D.W., Luo S. (2010). Determination the modifier content in SBS modified asphalt based on infrared spectroscopy technique. Appl. Mech. Mater..

[24] Hou X., Lv S., Chen Z., Xiao F. (2018). Applications of Fourier transform infrared spectroscopy technologies on asphalt materials. Measurement.

[25] Sun D.Q., Zhang L.W., Zhang X.L. (2011). Quantification of SBS content in SBS polymer modified asphalt by FTIR. Adv. Mater. Res..

[26] Li Y., Wu S., Pang L., Liu Q., Wang Z., Zhang A. (2018). Investigation of the effect of Mg-Al-LDH on pavement performance and aging resistance of styrene-butadiene-styrene modified asphalt. Constr. Build. Mater..

[27] Xue Y., Hu Z., Wang C., Xiao Y. (2019). Evaluation of dissolved organic carbon released from aged asphalt binder in aqueous solution. Constr. Build. Mater..

[28] Misra A., Poorsolhjouy P. (2016). Granular micromechanics based micromorphic model predicts frequency band gaps. Contin. Mech. Thermodyn..

[29] Nejadsadeghi N., Placidi L., Romeo M., Misra A. (2019). Frequency band gaps in dielectric granular metamaterials modulated by electric field. Mech. Res. Commun..

[30] Li Y., Wu S., Liu Q., Nie S., Li H., Dai Y., Pang L., Li C., Zhang A. (2019). Field evaluation of LDHs effect on the aging resistance of asphalt concrete after four years of road service. Constr. Build. Mater..

[31] García Á. (2012). Self-healing of open cracks in asphalt mastic. Fuel.

[32] Ayar P., Moreno-Navarro F., Rubio-Gámez M.C. (2016). The healing capability of asphalt pavements: A state of the art review. J. Clean. Prod..

[33] Grossegger D., Garcia A. (2019). Influence of the thermal expansion of bitumen on asphalt self-healing. Appl. Therm. Eng..

[34] Liu Q., García Á., Schlangen E., van de Ven M. (2011). Induction healing of asphalt mastic and porous asphalt concrete. Constr. Build. Mater..

[35] Little D.N., Prapnnachari S., Letton A., Kim Y. (1993). Investigation of the Microstructural Mechanism of Relaxation and Fracture Healing in Asphalt.

[36] Lv Q., Huang W., Zhu X., Xiao F. (2017). On the investigation of self-healing behavior of bitumen and its influencing factors. Mater. Des..

